# The effect of tree age, daily sap volume and date of sap collection on the content of minerals and heavy metals in silver birch (*Betula pendula* Roth) tree sap

**DOI:** 10.1371/journal.pone.0244435

**Published:** 2020-12-29

**Authors:** Paweł Staniszewski, Maciej Bilek, Wojciech Szwerc, Robert Tomusiak, Paweł Osiak, Ryszard Kocjan, Tadeusz Moskalik

**Affiliations:** 1 Department of Forest Utilization, Institute of Forest Sciences, Warsaw University of Life Sciences–SGGW, Warsaw, Poland; 2 Department of Agroecology, Institute of Agricultural Sciences, Land Management and Environmental Protection, University of Rzeszów, Rzeszów, Poland; 3 Chemistry Department of Analytical Chemistry, Medical University of Lublin, Lublin, Poland; 4 Department of Forest Management Planning, Dendrometry and Forest Economics, Institute of Forest Sciences, Warsaw University of Life Sciences–SGGW, Warsaw, Poland; 5 Forestry Students’ Scientific Association, Warsaw University of Life Sciences–SGGW, Warsaw, Poland; West Virginia University, UNITED STATES

## Abstract

The aim of this study was to determine the effect of the age of trees, daily sap volume as well as the term of tapping birch sap collected in the forest environment on the content of selected minerals (zinc, copper and manganese) and heavy metals (lead, nickel, chromium and cadmium). The study was performed on material taken from two stands (aged 34 and 84 years) in a moist broadleaved forest habitat with a dominant share of silver birch (*Betula pendula* Roth).

The research results confirmed the presence of both nutritional essential minerals and hazardous heavy metals in the birch sap. At the same time, the content of minerals and heavy metals was found to be very variable and the differences between their concentrations, recorded on the same day of collecting in several trees of the same age group, can be even several dozen times higher. Depending on the examined elements, the factors influencing their content vary. The age of the trees determines only the manganese content; daily sap volume significantly affects the content of manganese and copper, and date of collection differentiates the content of zinc, lead, nickel and cadmium.

The results may be interesting in the context of developing procedures for collecting birch sap for the purpose of obtaining raw material with beneficial nutritional values and a high level of health safety. For this reason, our recommendation for guaranteeing the health safety and high nutritional value of birch sap is to combine batches of raw material taken from as many trees as possible, and at the same time to publicize the fact that collecting birch sap from just one single tree may result in a raw material that is both dangerous and has no nutritional benefits.

## Introduction

The use of non-wood forest resources, which for centuries was only a spontaneous human activity, is not only of historical significance today [1–4]. Hundreds of millions of people in the world still obtain a significant part of their basic, daily needs by using numerous forest resources [5,6]. Moreover, the collection, sale and processing of such resources are becoming a significant factor in stimulating regional development and improving the economic situation especially of poor rural communities [7,8]. The most frequently harvested forest resources primarily include mushrooms, forest fruits and medicinal plants [9,10]. Their value sometimes exceeds the importance of wood production, and harvesting is indicated as an opportunity to diversify the income of forest owners [11,12], especially in the face of climate change and the resulting consequences for forest management [13].

It is in this context that new opportunities for non-wood forest use are sought through research and the promotion and formalisation of new or currently forgotten forest resources [14]. Among them, birch sap is indicated as one of the most promising non-wood forest resources of central Europe, with very wide possibilities of its practical use, e.g. in the food and cosmetics industries [15–17]. The popularity of this forest product observed today is not only due to the unique role of birch trees in the culture and beliefs of the region [18,19], but also, and above all, to numerous ethnographic studies discussing the old traditional uses of birch sap as medicine product and a source of nutritional benefits [20–22].

The growing popularity of birch sap in central Europe is accompanied by increasing studies on the chemical composition and impact on the human health of this raw material obtained in the region [23–28]. In light of these studies, birch sap from central Europe can be considered primarily as a rich source of minerals, mainly copper, zinc and manganese. These minerals have a comprehensive impact on, among others, the human immune and reproductive systems and on the condition of skin, hair and nails [23,24,29,30]. At the same time, it was found that birch sap has fundamental restrictions, undermining its use as a potential raw material for the food industry. It is very susceptible to anthropopressure, which is particularly visible in the content of industrial and agricultural contaminants in birch sap: heavy metals, inorganic anions, residues of plant protection products and polycyclic aromatic hydrocarbons [30,31]. However, collecting birch sap from the forest environment, the least polluted ecosystem, solves these problems [32–34]. Another restriction relating to the nutritional properties of birch sap is its highly variable mineral composition, occurring both between trees and sites [24,29]. It is independent of the collecting site and also relates to the forest environment [35]. Large variation of mineral content was also found in the time profile, which additionally makes it difficult to obtain certain nutritional benefits from birch sap [29,36]. An analogous situation was found for the heavy metals content of birch sap. Their concentrations also greatly vary, both in the sap taken from adjacent trees and in the sap of the same tree, at one-day intervals, which subsequently necessitates the constant monitoring of the raw material collected [37]. Therefore, the aim of the current scientific studies should be to explain these phenomena and to search for factors other than those relating to the tree, site or time that affect the variability in the content of the mineral components and heavy metals in birch sap. For example, studies have been recently performed on the effect of tree DBH on the content of selected minerals and heavy metals in birch sap [38,39]. Only if a stable chemical composition is guaranteed through appropriate collecting procedures will it be possible to ensure a high nutritional value and, at the same time, the health safety of birch sap intended for the food industry and human consumption.

The aim of this study was to determine the effect of the age of trees, daily sap volume and date of sap collection from silver birch trees growing in a forest environment on the content of selected minerals and heavy metals. This will enable the development of the procedures of birch sap harvesting for the purpose of obtaining raw material with favourable nutritional values and a high level of health safety.

## Materials and methods

### Obtaining the study material

The research was conducted in central-eastern Poland, in the Siedlecka Upland mesoregion (Garwolin Forest District, Żelechów Forest District) of the Mazovian-Podlasie region. The research material was collected from two areas (units 43c and 44c), in the same habitat (moist broadleaved forest), with the similar degree of moisture (related to the forest habitat) and the similar proportion of the dominant species − silver birch (*Betula pendula* Roth.), while the age range was 84 and 34 years respectively. Other general characteristics of forest stands are as follows. The area of unit 43c is 8.00 ha, average DBH—33 cm, average. height—25 m, share of silver birch– 50% and GPS coordinates—51.7542; 21.9885. Area of unit 44c is 2.04 ha, avg. DBH—19 cm, avg. height—17 m, share of silver birch– 60% and GPS coordinates—51.7640; 21.9275 [40]. According to the data from the last 30 years, the average annual temperature in the study area is 8.9°C, and the average annual precipitation is 516 mm [41]. The Hartig’s method [42] was used to designate sample trees representing the stand. This method is based on taking trees ordered by increasing diameter at breast height (DBH) and categorizing them into three classes of the same cross-sectional area. Then, two sample trees of average DBH value from each class were selected. For the 34-year-old stand, these values were: 14, 21 and 27 cm, whereas in the case of 84-year-old stand − 27, 35 and 39 cm. Healthy trees with well-developed crowns and without visible defects (6 trees from each stand) were selected for the study. From the beginning of March, air temperature was observed; when it remained at 8–10°C for several consecutive days, test drilling was performed to determine the beginning of sap flow. A hole of 1 cm diameter and 5 cm depth was drilled at a height of 1 m in each tree on its southern side. The hole was then connected to a closed storage container with a plastic pipe. Sap was collected from each tree for 24 hours, three times, at weekly intervals: March 12 (collection date I), March 18 (date II) and March 25, 2017 (date III). The three dates correspond to the successive phases of the sap leak. In each case, the daily volume of sap was determined and a sample was taken for chemical tests, which was immediately frozen.

### Zn, Cu and Mn determination using the flame technique of atomic absorption spectrometry (F-AAS) method

#### Sample preparation

Stock solutions at a concentration of 1.0 ppm (mg/l) for zinc, 1.5 ppm for copper, 1.5 ppm for manganese were prepared by dilution the standard solutions at a concentration of 1000 ppm (Merck. Germany) in 0.5% nitric acid; prepared by diluting 65% Suprapur nitric acid (Merck, Darmstadt, Germany) in deionized water by Ultrapure Millipore Direct-Q-R 3UV (Merck, Darmstadt, Germany) with a resistivity of 18.2 MΩ∙cm.

#### Optimization of F-AAS parameters

In order to select the right analysis conditions in the flame technique, the following parameters were optimized: burner flame height and gas (air–acetylene) flow rate. In addition, an ionizing buffer of 0.1% potassium chloride solution was used. The most optimal parameters are summarized in Table 1.

**Table 1:** Optimized parameters of the F-ASS technique.

Element	Burner height (mm)	Flow rate (l/h)
Zn	5	50
Cu	6	50
Mn	5	45

#### Atomic absorption spectrometry analysis methods

The contents of the analysed trace elements were determined by the flame technique of atomic absorption spectrometry using the High-Resolution Continuum Source Atomic Absorption Spectrometer ContrAA 700 (Analytik, Jena, Germany). The calibration curves for the elements were selected by the decomposition of residues method. 0.1% KCl as an ionization buffer was added to calibration standard solutions and each sample. The dilution factor was chosen automatically by the autosampler (Table 2).

**Table 2:** Validation parameters of the analytical method using the F-AAS technique.

Element	Line (nm)	Conc. range (mg/l)	Correlation coeff. (R)	Precision (%RSD)	LOD (mg/l)	LOQ (mg/l)
Zn	213.857	0–1	0.9999	0.5–1.6	0.0165	0.0880
Cu	324.754	0–1.5	0.9998	0.3–1.1	0.0093	0.0511
Mn	279.482	0–3.0	0.9999	1.9–2.7	0.0081	0.0440

### Pb, Ni, Cr and Cd determination using the graphite-furnace atomic absorption spectrometry (GF-AAS)

#### Sample preparation

Stock solutions at concentration of 3.0 ppb (μg/l) for cadmium, 35 ppb for chromium, 60 ppb for nickel and 30 ppb for lead were prepared by dilution the standard solutions at a concentration of 1000ppm (Merck, Darmstadt, Germany) in 0.5% nitric acid; prepared by diluting 65% Suprapur nitric acid (Merck, Darmstadt, Germany) in deionized water by Ultrapure Millipore Direct-Q-R 3UV (Merck, Darmstadt, Germany) with a resistivity of 18.2 MΩ∙cm.

#### Optimization of GF-AAS parameters

In order to select the appropriate pyrolysis and atomization temperatures of the analysed elements by the electrothermal method, these processes were optimized. At the same time, we examined whether there was a need for a palladium-magnesium or magnesium matrix modifier during the quantitative analysis. In all cases of quantitative determination, the use of a magnesium matrix modifier in the form of 0.1% magnesium nitrate significantly improved the precision of the analytical method (Table 3).

**Table 3:** Optimized time-temperature parameters in the GF-AAS technique.

Element	Drying temp. [°C]	Pyrolysis temp. [°C]	Atomization temp. [°C]
Pb	110	900	1900
Ni	110	1050	2300
Cr	110	1300	2300
Cd	110	700	1600

#### Atomic absorption spectrometry analysis methods

The contents of the analysed trace elements were determined by the electrothermal technique of atomic absorption spectrometry with atomization in a L’vov platform graphite cuvette using the High-Resolution Continuum Source Atomic Absorption Spectrometer ContrAA 700 (Analytik, Jena, Germany). The calibration curves for the elements were selected by the decomposition of residues method. The dilution factor of the analysed samples was chosen automatically by the autosampler. 20 μl of each sample solution with 5 μl of 0.1% Mg(NO_3_)_2_ matrix modifier was injected on the cuvette platform (Table 4).

**Table 4:** Validation parameters of the analytical method using the GF-AAS technique.

Element	Line (nm)	Conc. range (μg/l)	Correlation coeff. (R)	Precision (%RSD)	LOD (μg/l)	LOQ (μg/l)
Pb	217.00	0–30.0	0.9999	0.6–1.4	0.9899	1.1280
Ni	232.00	0–60.0	0.9999	0.8–2.2	0.4250	1.3380
Cr	357.87	0–35.0	0.9999	0.5–6.4	0.1510	0.5210
Cd	228.80	0–3.0	0.9999	0.6–1.5	0.0588	0.2409

### Statistical analysis

Since the content of some of the elements in the tested birch sap samples was below the limit of quantification (LOQ), in order to calculate the average values (mean, median), the measurement results were set at half of the LOQ value for a given element. If the average values calculated in this way were below the limit of quantification, an annotation of “below the limit of quantification” was added to the results. The approach adopted is analogous to Commission Directive 2009/90/EC of 31 July 2009 (Article 5) laying down, pursuant to 2000/60/EC of the European Parliament and of the Council, technical specifications for chemical analysis and monitoring of water status [43].

We used the general linear model (GLM) for comparing how several variables (categorical ones: age, sap collection date, and a continuous one: daily sap volume) as well as the interactions affecting the content of the elements (Zn, Cu, Mn, Pb, Ni, Cd, excluding Cr—see results) in birch sap (continuous variables). GLM strictly assumes that the model residuals follow a conditionally normal distribution. This assumption was checked with the Kolmogorov–Smirnov test at a 0.05 significance level. This assumption was met for Zn, Pb, Cd. In the case of elements for which the rest of the model did not obtain a normal distribution, Box-Cox Transformations were performed. This was done for the content of Cu, Mn, Ni. The general linear models were performed in R software with the stats package, and the figures were done with the graphics and sjPlot packages. The results of GLM were presented graphically showing the content of the elements in birch sap only for those factors for which GLM demonstrated a significant impact. Moreover, all variables were characterized using measures of descriptive statistics.

## Results

**Fig 1:**
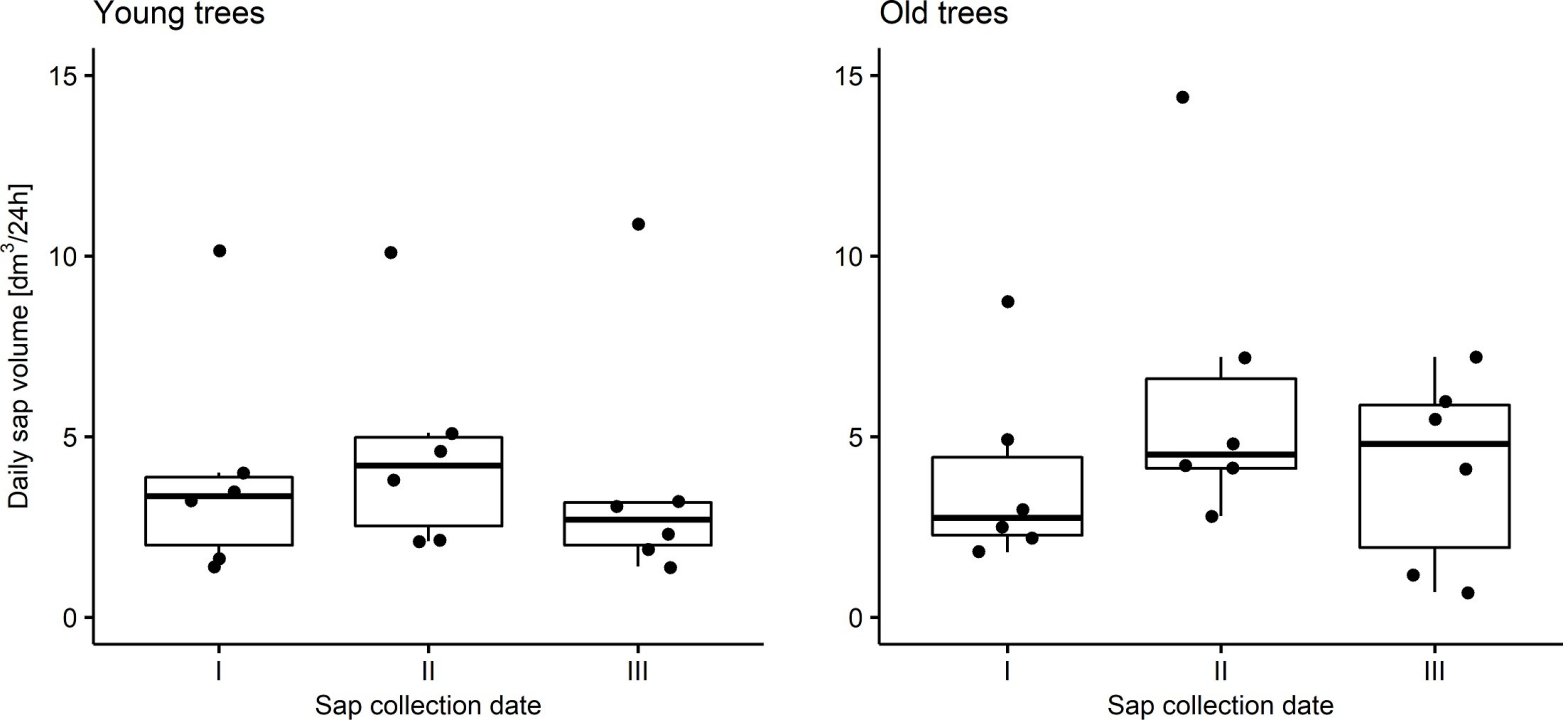
Distributions of daily sap volume depending on sap collection date (I-III) for young and old trees (box: 25th percentile (Q1), median and 75th percentile (Q3); whiskers: min. value within Q1-1,5*IQR and max value within Q3+1,5*IQR, circles: outliers).

Daily sap volume had values of from 1.40 dm^3^ to 10.90 dm^3^ in the younger group of trees, with an average value of 4.13 dm^3^ and a standard deviation of 3.06 dm^3^ (Table 5). In the group of older trees, a wider range of variability in this feature was observed: the minimum was 0.70d dm^3^/24h and the maximum was 14.40 dm^3^/24h. The average daily sap volume of older trees was 4.74 dm^3^ with a standard deviation of 3.26 dm^3^. Taking into account the sap collection dates, the highest arithmetic means were observed at the second date, both in the group of younger and older trees. The lowest average values in the group of younger trees were observed at date III, and in the older trees—at date I. The highest variability of the daily sap volume of the younger trees was observed at the third date (standard deviation 3.55 dm^3^/24h), while of older trees—at the second date (4.25 dm^3^/24h). Detailed summary statistics of daily sap volume in a group of younger and older trees at different sap collection dates is presented in Table 5. Comparison of distributions of daily sap volume for each group is presented at Fig 1.

**Table 5:** Summary statistics for daily sap volume (dm^3^/24h).

Sap collection date	Number of observations	Mean	Standard deviation	Median	Lower quartile	Upper quartile	Minimum	Maximum
**young trees**								
I	6	3,97	3,18	3,35	1,60	4,00	1,40	10,10
II	6	4,63	2,95	4,20	2,10	5,10	2,10	10,10
III	6	3,80	3,55	2,70	1,90	3,20	1,40	10,90
**Total**	**18**	**4,13**	**3,06**	**3,20**	**2,10**	**4,60**	**1,40**	**10,90**
**old trees**								
I	6	3,85	2,61	2,75	2,20	4,90	1,80	8,70
II	6	6,25	4,25	4,50	4,10	7,20	2,80	14,40
III	6	4,12	2,65	4,80	1,20	6,00	0,70	7,20
**Total**	**18**	**4,74**	**3,26**	**4,15**	**2,50**	**6,00**	**0,70**	**14,40**

**Fig 2:**
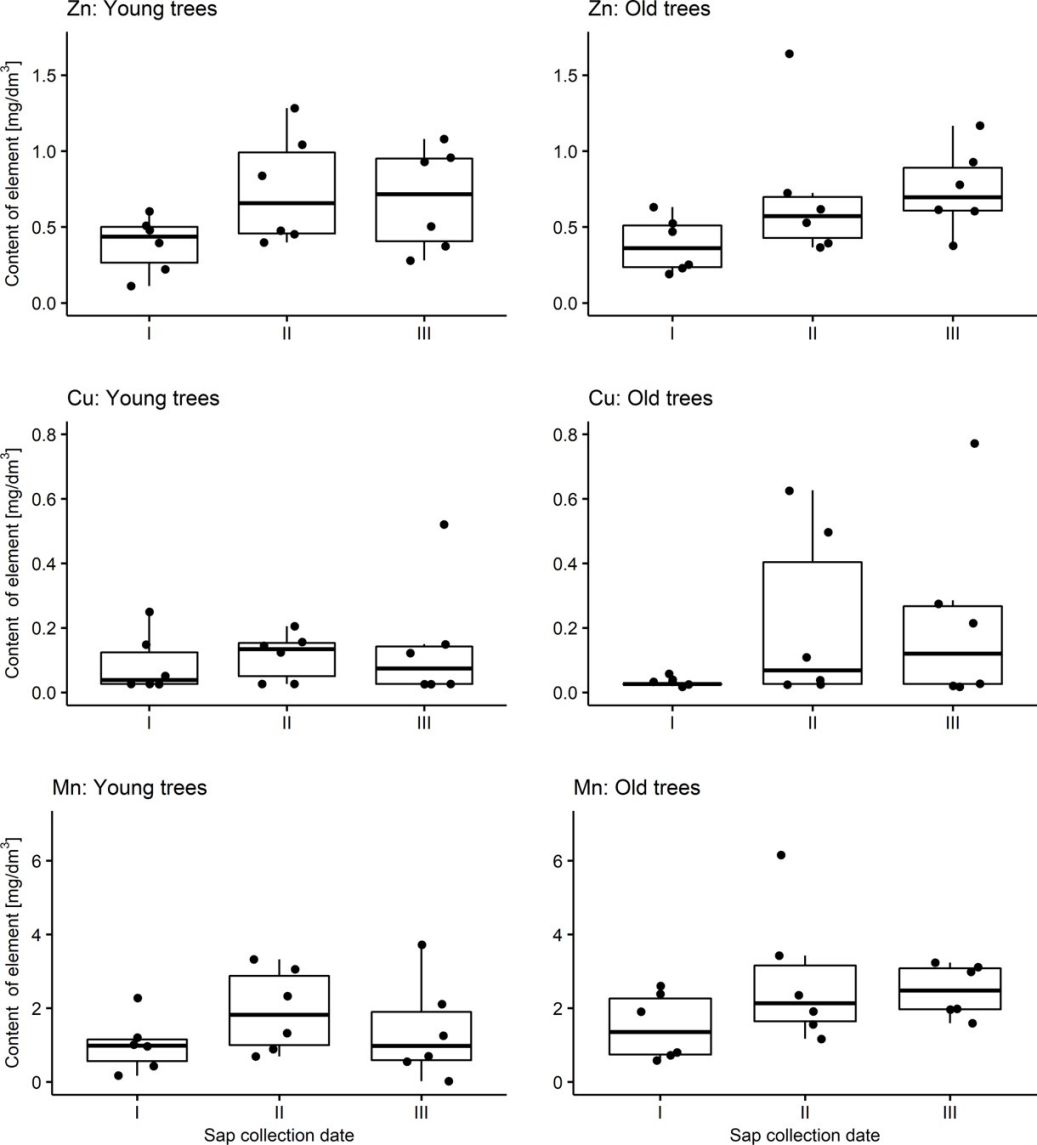
Distributions of the contents of minerals in birch sap for young and old trees depending on the sap collection date (box: 25th percentile (Q1), median and 75th percentile (Q3); whiskers: min. value within Q1-1,5*IQR and max value within Q3+1,5*IQR).

**Fig 3:**
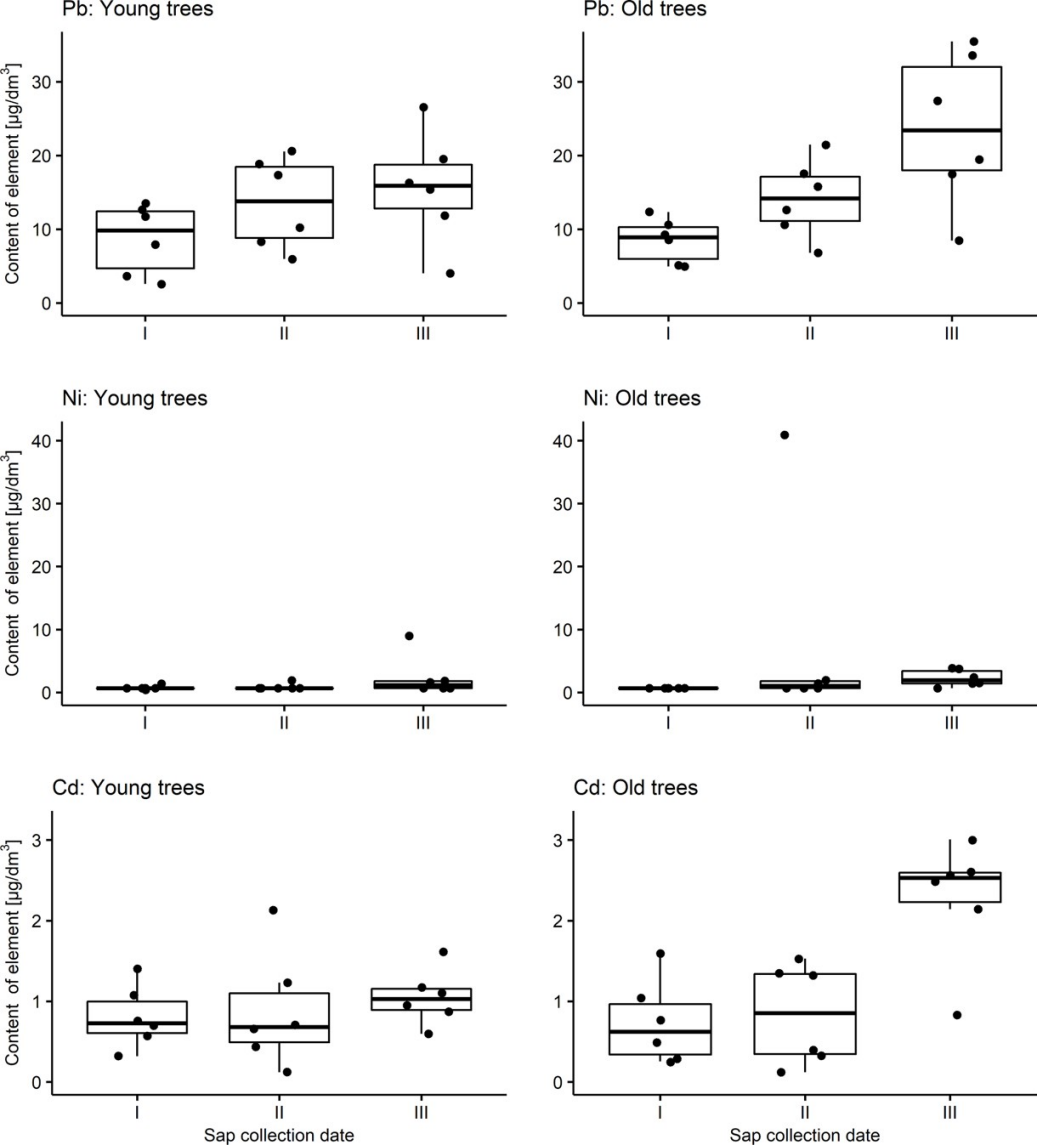
Distributions of the contents of heavy metals in birch sap for young and old trees depending on the sap collection date (box: 25th percentile (Q1), median and 75th percentile (Q3); whiskers: min. value within Q1-1,5*IQR and max value within Q3+1,5*IQR).

The content of elements in birch sap from younger and older trees was examined. The measures of the descriptive statistics for each group are presented in Table 6 (minerals) and Table 7 (heavy metals). These values for Zn were on average 0.61 mg/dm^3^ both in younger and older trees, while for Mn they were 1.45 for young trees and 2.25 mg/dm^3^ for old ones. Respectively, average values for heavy metals were: Pb 12.60 and 15.40 μg/dm^3^, Ni 1.34 and 3.55 μg/dm^3^, Cd 0.91 and 1.28 μg/dm^3^. A comparison of the average content of particular elements in birch sap from younger and older trees was not possible in several cases. For Cu, the average value in the group of younger trees was 0.12 mg/dm^3^, for older trees it was 0.16 mg/dm^3^; for Ni 1.34 μg/dm^3^ and 3.55 μg/dm^3^ respectively. All values for Cr were below LOQ. The distributions of the contents of individual elements are presented in Fig 2 (minerals) and Fig 3 (heavy metals).

**Table 6:** Summary statistics for the content of minerals in birch sap (mg/dm^3^).

Age	Sap collection date	Number of: values below LOQ/all measurements	Mean	Standard deviation	Median	Lower quartile	Upper quartile	Minimum	Maximum
**Zn**									
young trees	I	0/6	0,39	0,19	0,44	0,22	0,51	0,11	0,60
	II	0/6	0,75	0,36	0,66	0,45	1,04	0,40	1,28
	III	0/6	0,69	0,34	0,72	0,37	0,96	0,28	1,08
	**total**	**0/18**	**0,61**	**0,33**	**0,49**	**0,40**	**0,93**	**0,11**	**1,28**
old trees	I	0/6	0,38	0,18	0,36	0,23	0,52	0,19	0,63
	II	0/6	0,71	0,47	0,57	0,39	0,72	0,37	1,64
	III	0/6	0,75	0,28	0,70	0,61	0,93	0,38	1,17
	**total**	**0/18**	**0,61**	**0,36**	**0,57**	**0,38**	**0,72**	**0,19**	**1,64**
**Cu**									
young trees	I	3/6	0,09	0,09	0,04*	0,03*	0,15	0,03*	0,25
	II	2/6	0,11	0,07	0,13	0,03*	0,16	0,03*	0,20
	III	3/6	0,15	0,19	0,07	0,03*	0,15	0,03*	0,52
	**total**	**8/18**	**0,12**	**0,12**	**0,09**	0,03*	**0,15**	0,03*	**0,52**
old trees	I	5/6	0,03*	0,01*	0,03*	0,03*	0,03*	0,03*	0,06
	II	3/6	0,22	0,27	0,07	0,03*	0,50	0,03*	0,63
	III	3/6	0,23	0,29	0,12	0,03*	0,29	0,03*	0,78
	**total**	**11/18**	**0,16**	**0,24**	0,03*	0,03*	**0,21**	0,03*	**0,78**
**Mn**									
young trees	I	0/6	1,01	0,73	0,99	0,43	1,20	0,17	2,27
	II	0/6	1,93	1,13	1,82	0,89	3,06	0,69	3,33
	III	1/6	1,39	1,34	0,98	0,56	2,11	0,02*	3,72
	**total**	**1/18**	**1,45**	**1,10**	**1,11**	**0,69**	**2,27**	0,02*	**3,72**
old trees	I	0/6	1,50	0,90	1,35	0,72	2,38	0,58	2,60
	II	0/6	2,76	1,83	2,14	1,56	3,43	1,17	6,15
	III	0/6	2,48	0,71	2,48	1,96	3,11	1,59	3,24
	**total**	**0/18**	**2,25**	**1,30**	**1,97**	**1,56**	**2,98**	**0,58**	**6,15**

* values below LOQ (Zn—0,088 mg/dm^3^, Cu—0,0511 mg/dm^3^, Mn—0,044 mg/dm^3^).

**Table 7:** Summary statistics for the content of heavy metals in birch sap (μg/dm3).

Age	Sap collection date	Number: values below LOQ/all measurements	Mean	Standard deviation	Median	Lower quartile	Upper quartile	Minimum	Maximum
**Cr**									
young trees	I	6/6	0,26*	0,00	0,26*	0,26*	0,26*	0,26*	0,26*
	II	6/6	0,26*	0,00	0,26*	0,26*	0,26*	0,26*	0,26*
	III	6/6	0,26*	0,00	0,26*	0,26*	0,26*	0,26*	0,26*
	**total**	**18/18**	0,26*	**0,00**	0,26*	0,26*	0,26*	0,26*	0,26*
old trees	I	6/6	0,26*	0,00	0,26*	0,26*	0,26*	0,26*	0,26*
	II	6/6	0,26*	0,00	0,26*	0,26*	0,26*	0,26*	0,26*
	III	6/6	0,26*	0,00	0,26*	0,26*	0,26*	0,26*	0,26*
	**total**	**18/18**	0,26*	**0,00**	0,26*	0,26*	0,26*	0,26*	0,26*
**Pb**									
young trees	I	0/6	8,63	4,71	9,81	3,58	12,65	2,56	13,39
	II	0/6	13,53	6,12	13,80	8,30	18,84	5,95	20,52
	III	0/6	15,64	7,54	15,91	11,90	19,52	4,02	26,57
	**total**	**0/18**	**12,60**	**6,58**	**12,28**	**7,97**	**17,34**	**2,56**	**26,57**
old trees	I	0/6	8,46	2,97	8,90	5,09	10,60	4,93	12,33
	II	0/6	14,12	5,23	14,19	10,58	17,54	6,78	21,44
	III	0/6	23,63	10,37	23,42	17,46	33,55	8,47	35,45
	**total**	**0/18**	**15,40**	**9,15**	**12,47**	**8,54**	**19,45**	**4,93**	**35,45**
**Ni**									
young trees	I	5/6	0,74*	0,32	0,67*	0,67*	0,67*	0,39*	1,36
	II	5/6	0,87*	0,49	0,67*	0,67*	0,67*	0,67*	1,88
	III	3/6	2,41	3,27	1,14*	0,67*	1,85	0,67*	9,00
	**total**	**13/18**	**1,34**	**1,96**	0,67*	0,67*	**1,36**	0,39*	**9,00**
old trees	I	6/6	0,67*	0,00	0,67*	0,67*	0,67*	0,67*	0,67*
	II	3/6	7,71	16,28	1,04*	0,67*	1,93	0,67*	40,92
	III	1/6	2,26	1,31	1,95	1,41	3,75	0,67*	3,84
	**total**	**10/18**	**3,55**	**9,38**	0,67*	0,67*	**1,93**	0,67*	**40,92**
**Cd**									
young trees	I	0/6	0,80	0,38	0,73	0,57	1,08	0,32	1,40
	II	1/6	0,88	0,71	0,68	0,44	1,23	0,12*	2,13
	III	0/6	1,05	0,34	1,03	0,87	1,17	0,60	1,62
	**total**	**1/18**	**0,91**	**0,49**	**0,81**	**0,60**	**1,17**	0,12*	**2,13**
old trees	I	0/6	0,73	0,51	0,62	0,29	1,03	0,25	1,59
	II	1/6	0,84	0,62	0,85	0,33	1,35	0,12*	1,53
	III	0/6	2,27	0,76	2,53	2,14	2,60	0,84	3,01
	total	**1/18**	1,28	0,94	1,17	0,40	2,14	0,12*	3,01

* values below LOQ (Cr—0,521 μg/dm^3^, Pb—1,128 μg/dm^3^, Ni—1,338 μg/dm^3^, Cd– 0,241 μg/dm^3^).

Taking into account the sap collection dates, the highest average value of Zn content in birch sap taken from younger trees was found at date II; this date also had the greatest variability in the content of this element. In the group of older trees, the highest average Zn content was found at date III, and the highest variability–at date II. In the case of Cu, the highest average content and the highest variability in both younger and older trees were observed at date III. Mn content in the group of younger trees had the highest average at date II, while the highest variability at date III. In the group of older trees, both the highest average content of this element and the highest variability were found at the second date (Table 6).

Of the heavy metals, no analysis could be performed for Cr, because all its values were lower than LOQ. Pb content turned out to be the highest at the collection date III, both in the group of younger and older trees. This term also exhibited the largest variability of trees in terms of the content of this element in birch sap for both age groups. In the case of Ni in the group of young trees, both the highest content and the greatest variability of this feature were found at date III. On the other hand, both measures (mean and standard deviation) in the group of older trees had the highest values at the second measurement date. Average Cd content in birch sap turned out to be the highest at date III in both younger and older trees. At this date, the highest variability of Cd content was found in older trees. In the case of the group of younger trees, the greatest variation in the value of this feature was found at date II (Table 7).

**Fig 4:**
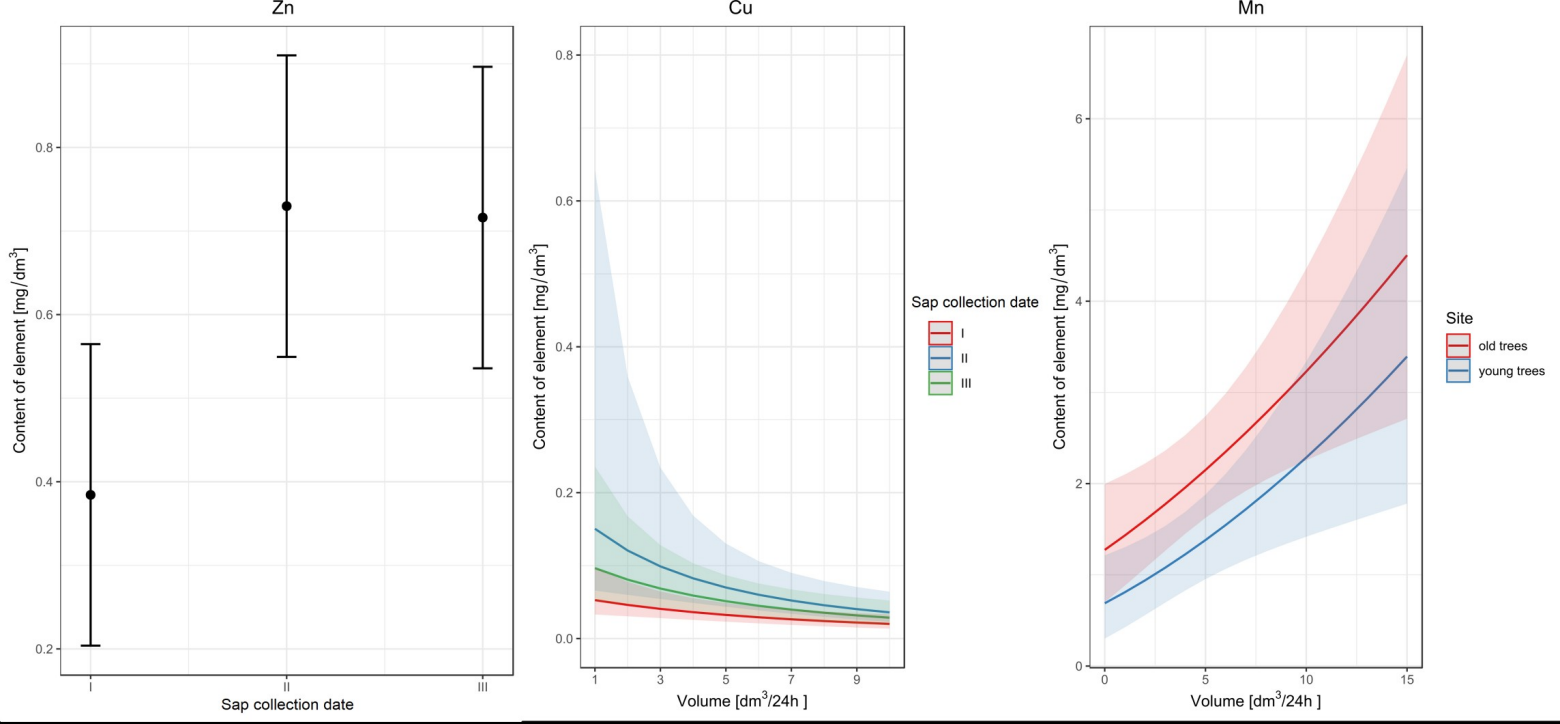
Effect of different factors on the content of minerals in birch sap.

Using GLM, we checked which factors (age, sap collection date, daily sap volume) affect the content of selected elements in birch sap. The impact of these factors varies depending on the element studied (Tables 8 and 9). In the case of Zn, the sap collection date proved to be an important factor (Table 8); in the first term, the content of this element in birch sap was significantly lower than in terms II and III, constituting one homogeneous group (Fig 4).

**Table 8:** Prediction of the content of minerals in birch sap by the GLM (mg/dm^3^).

Predictors	Zn			Cu*			Mn*		
	Estimates	CI	p	Estimates	CI	p	Estimates	CI	p
(Intercept)	0.38	(0.20, 0.50)	**<0.001**	-6.17	(-8.82, -3.52)	**<0.001**	0.26	(-0.32, 0.84)	0.373
Sap collection date [II]	0.35	(0.09, 0.60)	**0.009**	3.61	(0.55, 6.66)	**0.022**			
Sap collection date [III]	0.33	(0.08, 0.59)	**0.012**	2.31	(-0.68, 5.30)	0.125			
Daily sap volume				-0.61	(-1.02, -0.20)	**0.005**	0.14	(0.05, 0.23)	**0.004**
Age [young trees]							-0.60	(-1.15, -0.04)	**0.037**
Observations		36			36			36	
R^2^/R^2^ adjusted	0.228/0.181			0.291/0.225			0.325/0.284		
F-statistic/overall p	4.869/**0.014**			4.384/**0.011**			7.952/**0.002**		

* Values after Box-Cox transformations.

CI– 95% confidence interval, p–probability value (p-value), R^2^ –coefficient of determination.

Bolded p-values indicate significant impact of the predictor on the content of elements at α = 0.05.

**Table 9:** Prediction of the content of heavy metals in birch sap by the GLM (μg/dm^3^).

Predictors	Pb			Ni*			Cd		
	Estimates	CI	p	Estimates	CI	p	Estimates	CI	p
(Intercept)	8.55	(4.60, 12.50)	**<0.001**	-0.51	(-0.80, -0.22)	**0.001**	0.73	(0.25, 1.21)	**0.004**
Sap collection date [II]	5.28	(-0.31, 10.87)	0.063	0.37	(-0.04, 0.79)	0.075	0.10	(-0.57, 0.78)	0.755
Sap collection date [III]	11.09	(5.50, 16.67)	**<0.001**	0.72	(0.31, 1.14)	**0.001**	1.54	(0.86, 2.22)	**<0.001**
Interactions: Sap collection date [I]: Age [young trees]							0.07	(-0.61, 0.75)	0.835
Sap collection date [II]: Age [young trees]							0.04	(-0.64, 0.72)	0.900
Sap collection date [III]: Age [young trees]							-1.22	(-1.90, -0.54)	**0.001**
Observations		36			36			36	
R^2^/R^2^ adjusted	0.331/0.290			0.277/0.233			0.509/0.427		
F-statistic/overall p	8.155/**0.001**			6.314/**0.005**			6.224/**<0.001**		

* Values after Box-Cox transformations.

CI– 95% confidence interval, p–probability value (p-value), R^2^ –coefficient of determination.

Bolded p-values indicate significant impact of the predictor on the content of elements at α = 0.05.

Two factors have a significant impact on the Cu content in birch sap: sap collection date and daily sap volume (Table 8). Comparison of the model curves presenting the relationship between the content of Cu and daily sap volume shows a trend in which the content of this element decreases as the daily birch sap volume increases. The location of the curves relative to each other indicates that the model has the highest Cu content at date II and the lowest at date I (Fig 4).

GLM results indicate that the Mn content in birch sap is significantly influenced by two factors: daily birch sap volume and age of trees (Table 8). Along with the increase in daily sap volume, the manganese content in birch sap increases, while this relationship is at a significantly higher level in the group of older trees (Fig 4).

**Fig 5:**
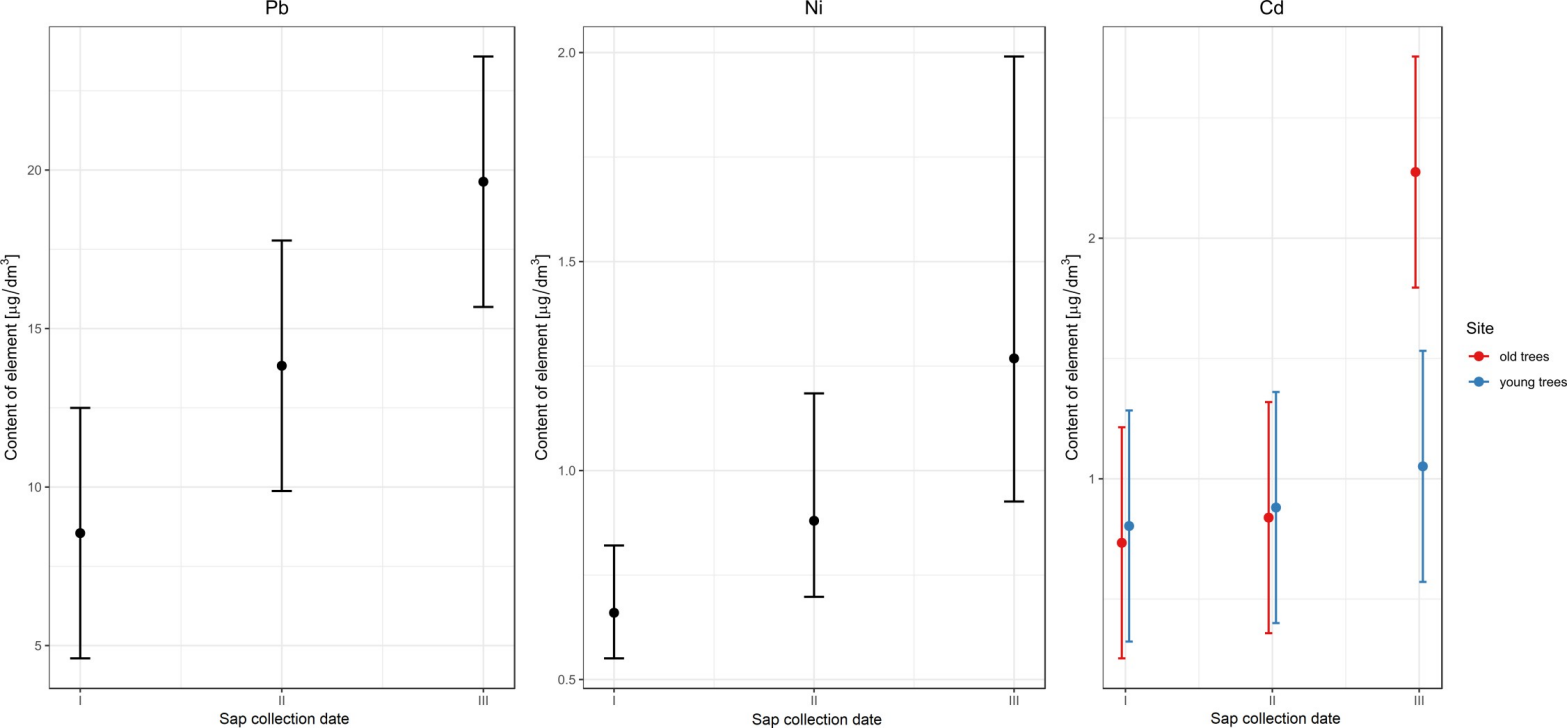
Effect of different factors on the content of heavy metals in birch sap.

GLM indicated the date of collection as a factor significantly affecting the content of Pb and Ni in birch sap (Table 9). The model shows a significant difference between dates I and III, in which the content of both Pb and Ni in birch sap is the highest (Fig 5).

According to GLM results, the sap collection date and interactions between the collection date and the age of the trees affect the content of Cd in birch sap (Table 9). At the first and the second dates, the Cd content is at the same level in the groups of younger and older trees. On the other hand, at date III, a significantly higher Cd content was found in the older stand (Fig 5).

## Discussion

The chemical composition of birch sap is very varied, both between trees and over time [35,36]. This was confirmed in our study, in which both minerals and heavy metal concentrations differed within one collection period and between six trees in the same age group several, a dozen or even several dozen times. The extreme situation was observed for the concentration of manganese in the third birch tapping, where the difference between the highest and the lowest concentration was almost 170 times. Depending on the element, the factors affecting their content in birch juice turned out to be different. In the case of Zn, the only factor significantly affecting its content in birch sap was the date of collecting. GLM showed two factors for copper and magnesium. In the first case, it was collection date and daily sap volume; in the second, daily birch sap volume and age of trees.

Such a situation makes it difficult, if not impossible, to obtain not only certain nutritional benefits, but also to guarantee toxicological safety when consuming birch sap from just one tree. For this reason, the purpose of this study was to explain whether the observed variability can be linked to the age of the trees and the volume of raw material collected.

The age of a tree is a factor determining a number of utility parameters in forest management, including those relating to the chemical composition of wood [44]. In previous studies on birch sap, it was determined that the age of a tree influences the chemical composition of tree sap in the case of the content of nutritionally beneficial phenolic compounds [25]. The observed correlation was positive, i.e. the older the specimens of eight tree species, including silver birch, the richer the sap was in phenolic compounds. These findings, however, are not important practically because the concentrations of nutritionally beneficial phenolic compounds in the trees’ sap were many times lower than in typical food items, e.g. fruit and vegetable juices or herbal infusions [25].

Of greater importance seem to be the results of pilot studies investigating the correlation of the age of eight tree species with six minerals. The highest correlation was found for the content of zinc in black walnut sap (*Juglans nigra* L) as well as calcium and magnesium in box elder sap (*Acer negundo* L) [32], i.e. species introduced to central Europe, found only in parks, and not in forest habitats, where they could be used for tree sap collection. In this study, work was conducted on a species commonly found naturally, i.e. the silver birch (*Betula pendula* Roth) [45]. However, we did not find that the age of this tree species influenced the content of the minerals and heavy metals in sap. From the point of view of the process of collecting raw material, this is information of great practical significance. This means that, due to the utility values of the sap and its volume, the age of a tree does not have to be taken into account in the mass harvesting of sap for food processing. On the other hand, in the context of the rules of making trees and stands available for commercial sap harvesting in sustainable forest management [12,46], age should be taken into account, as only stands of cutting age should be treated as the source of the raw material for sap harvesting on an industrial scale.

The volume of birch sap obtained is a factor determining the profitability of its processing by the food industry. In central Europe, the daily volume of birch sap has been studied many times and is usually several litres per day [31,36,47,48]. As with the chemical parameters, the volume of collected birch sap is also variable, both between trees and over time, and in central Europe, it is significant that over the course of several weeks the volume of sap initially increases, reaches its maximum and then decreases until the flow disappears [36,48]. As a consequence, the high variability in the daily volume of birch sap can affect not only into concentrations of nutritionally beneficial minerals, but also of heavy metals, thus influencing the toxicological properties of the sap, all the more so as this raw material has already been found to be highly susceptible to contamination with these environmental toxins [32,37].

In the present study, concentrations of heavy metals, dangerous for human health, were found to be independent of the volume of birch sap collected. Therefore, from a toxicological point of view, there is no reason for the health safety of birch sap to be made dependent on its volume, which is subject to considerable fluctuations [32,36]. However, the results indicate a need to introduce recommendations of the time of harvesting: due to the higher concentrations of unfavourable ingredients, harvesting in the last period of sap flow should be avoided.

The volume of birch sap collected translates into nutritional properties. As we have established, the sap taken during a more intense flow contains more manganese, while that taken during a less intense flow contains more copper. In this study, however, we observed that at the same time, not only did the concentrations of mineral components differ among trees, but simultaneously, the volume of the birch sap obtained, i.e. in one day, less sap volume in some trees and more in others. For example, in the first birch tapping, 1.4 to 10.1 litres of sap were obtained from trees in the same age group. The combination of sap collected from many trees will therefore result in an averaging of the minerals concentrations and increase the likelihood of obtaining a stable composition of the raw material for the food industry [36,37]. By analogy, we can state that the currently promoted practice in social media of collecting birch sap from only one tree may result in obtaining a raw material of very low nutritional value on the one hand, and a high degree of health risk on the other. Meanwhile, birch sap collected en masse and processed by the food industry is characterized by averaged properties, both in terms of nutritional value and consumer health safety [49].

**Fig 6:**
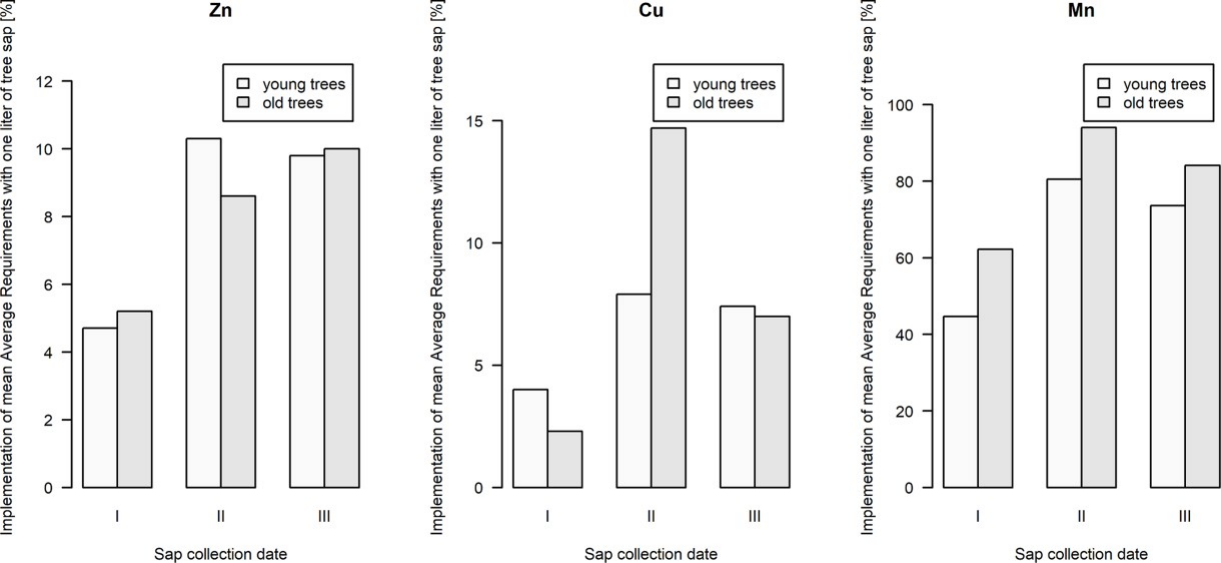
Implementation of daily dietary reference values (as recommended by EFSA for an adult woman) by one liter of birch sap obtained by combining the birch sap from 6 trees of one age group on successive collection dates.

The procedure of continuously collecting birch sap used in this study is of great importance for evaluating previous scientific research on this raw material. In these works [29,35,50], the methodology for testing the chemical composition of birch sap was based on collecting a specific, small volume of sap at the same time intervals, e.g. daily. In our study, a 24-hour collection process was used, allowing to characterize the collected batches by an averaged values. Additionally combining them and obtaining batches of several dozen litres of sap further eliminated the significant differences found in previous studies in the composition of birch sap between trees, with which nutritional restrictions and toxicological risks were associated [35,37]. An analogous method of sap collection was used to study the influence of tree DBH on the content of selected minerals and heavy metals in birch sap. However, no effect of tree diameter on the content of the studied elements was found [38,39]. The application of this method of sap collection for research make it possible to prove that the raw material formed by combining birch sap from many trees is still a rich source of copper, zinc and manganese. One litre of sap obtained by combining the 24-hour volume of six trees provided from several to several dozen percent of the daily dietary reference values recommended by the European Food Safety Authority [51–53], wherein the nutritional benefits from the content of copper and zinc were the lowest and manganese the highest (Fig 6).

Combining the sap taken from many trees is also of toxicological importance. Thanks to this procedure heavy metals concentration recorded in previous studies [24,54] are also averaged, thus the health risk that would occur when consuming the sap of just one tree is minimising. For example, a very high nickel concentration of 40.92 μg/dm^3^ was recorded in the sample taken from tree no. 6 in the older tree group on the second collecting term, regardless of the collection from a safe forest environment. However, after combining the daily sap volume of six trees from the same age group, the concentration of this heavy metal decreases to 16.46 μg/dm^3^. Therefore, both in the context of nutritional benefits and consumer health risks, the superiority of bottled saps offered by the food industry over sap taken individually from just one tree was confirmed [49].

**Fig 7:**
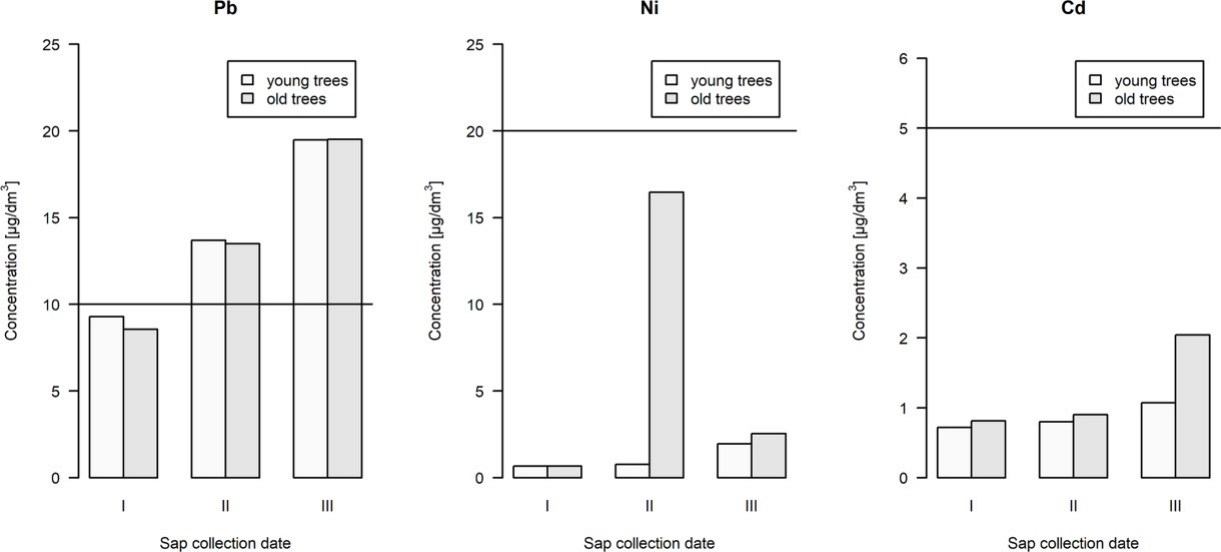
Concentration of heavy metals in birch sap obtained by combining the birch sap collected from 6 trees of one age group on successive collection dates, referenced to the parametric values for drinking water (horizontal line: guideline values for chemicals that are of health significance in drinking-water).

There are no food standards taking into account the health safety parameters of birch sap. However, the results obtained can be referenced to the parameters of food items with the most similar consistency and intended use, i.e. drinking water [55]. From the point of view of cadmium and nickel content, the combined sap of six trees did not exceed the recommended values for drinking water (5 and 20 μg/dm^3^, respectively), but exceeded them for lead (standard 10 μg/dm^3^) in the case of second and third collection date (Fig 7). It should be noted, however, that the standards for drinking water, a dietary item consumed in the greatest quantities, are the most stringent. As mentioned, the drinking water standard for lead is 10 μg/dm^3^, while it is three times higher for most fruit juices and nectars (0.03 mg/kg wet weight), ten times higher for most vegetables and fruits (0,1 mg/kg wet weight), and for leafy vegetables–thirty times higher (0.3 mg/kg wet weight) [56].

In relation to these standards, in turn, the examined birch sap exhibits full health safety and it also seems that the previously postulated continuous monitoring of the food safety of birch sap intended for the production of bottled beverages is not necessary [37]. Albeit, in the context of the results obtained, it should be noted that the production of birch syrup is currently being proposed in several central European countries [17,31]. In this case, the birch sap examined in this paper, despite the fact that it was collected in a forest environment, could not be used as the raw material to produce birch syrup because the content of heavy metals would increase several dozen times at least and would not meet food safety standards, especially due to the lead content. Therefore, in the case of birch sap collecting for syrup production, regular monitoring of heavy metal concentrations should be conducted before the costly evaporation process in order to avoid financial losses [57].

The very large differences in the mineral composition of birch sap observed so far [24,29] have also been confirmed in this paper. This all results encourage the initiation of further studies, which on the basis of the genetic analysis of the species silver birch (*Betula pendula* Roth) [58] practised in forest research, would allow trees to be selected with the most beneficial parameters of birch sap, both in terms of nutritional value and toxicological parameters. Birches that grow rapidly and resist droughts have been cultured within a species in a similar way, obtained by self-pollination and the free pollination of mother trees [59]. Shaping the nutritional properties of birch sap may also consist in the selection of a collecting site taking into account the properties of the soil environment. It has been shown that this has an impact on the chemical composition of the sap [60]. So far, however, the best way to guarantee nutritional benefits and the toxicological safety of birch sap is to combine the raw material taken from as many trees as possible.

## Conclusions

Birch sap contains elements valuable for human health. The average zinc content in the tested material ranges from 0.38 mg/dm^3^ to 0.75 mg/dm^3^, which accounts for approx. 4.7% to approx. 9.3% of daily dietary reference values for this element. In the case of copper, the values are from 0.03 mg/dm^3^ to 0.23 mg/dm^3^ (respectively: 2.3% - 17.7% of daily dietary reference values), while for manganese, from 1.01 mg/dm^3^ to 2.76 mg/dm^3^ (43% - 120%).

In the vast majority of cases, the content of heavy metals in birch sap does not exceed toxicological standards for drinking water. However, due to the high lead and nickel content in some samples, it is necessary to monitor the content of these elements during industrial sap harvesting and processing before the costly evaporation process in order to avoid financial losses.

The age of the trees turned out to be a factor that significantly influenced only manganese content. In the group of older trees, the content of this element is significantly higher. In the interaction with the date of sap collecting, age also varies the cadmium content of birch sap. However, the age of the tree affects the nutritional and toxicological properties of birch sap only to a minor extent.

We found that the daily volume of birch sap significantly influenced the content of selected mineral components: a positive correlation with volume was found for manganese and a negative one for copper.

The date of sap collecting significantly affects the content of zinc and copper, but also of heavy metals (lead, nickel and cadmium) in birch sap: the later the sap is collected, the higher the content of these elements can be expected. Considering the above, it is worth limiting sap harvesting in the last phase of the sap flow period.

The way to guarantee the health safety and high nutritional value of birch sap is to combine batches of the raw material obtained from as many trees as possible. The popular current collection and consumption of sap from just one tree is associated with a potential health risk and the lack of a beneficial nutritional effect.

## Supporting information

S1 Data (XLSX)
